# Analyzing communication skills of Pediatric Postgraduate Residents in Clinical Encounter by using video recordings

**DOI:** 10.12669/pjms.336.13481

**Published:** 2017

**Authors:** Attia Bari, Rehan Ahmed Khan, Uzma Jabeen, Ahsan Waheed Rathore

**Affiliations:** 1 Dr. Attia Bari, MBBS, DCH, MCPS, FCPS. MHPE. Associate Professor of Pediatric Medicine, Department of Pediatric Medicine, The Children’s Hospital and The Institute of Child Health, Lahore, Pakistan; 2 Rehan Ahmed Khan, FCPS, FRCS, JM-HPE, MSc HPE. Professor of Surgery, Islamic International Medical College, Riphah University, Rawalpindi, Pakistan; 3 Dr. Uzma Jabeen, FCPS. Assistant Professor of Pediatric Medicine, Department of Pediatric Medicine, The Children’s Hospital and The Institute of Child Health, Lahore, Pakistan; 4 Ahsan Waheed Rathore, DCH, MRCP, MRCPCH, FRCP. Professor of Pediatric Medicine, Department of Pediatric Medicine, The Children’s Hospital and The Institute of Child Health, Lahore, Pakistan

**Keywords:** Communication skills, Nonverbal communication, Postgraduate residents, Video recording

## Abstract

**Objective:**

To analyze communication skills of pediatric postgraduate residents in clinical encounter by using video recordings.

**Methods:**

This qualitative exploratory research was conducted through video recording at The Children’s Hospital Lahore, Pakistan. Residents who had attended the mandatory communication skills workshop offered by CPSP were included. The video recording of clinical encounter was done by a trained audiovisual person while the resident was interacting with the patient in the clinical encounter. Data was analyzed by thematic analysis.

**Results:**

Initially on open coding 36 codes emerged and then through axial and selective coding these were condensed to 17 subthemes. Out of these four main themes emerged: (1) Courteous and polite attitude, (2) Marginal nonverbal communication skills, (3) Power game/Ignoring child participation and (4) Patient as medical object/Instrumental behaviour. All residents treated the patient as a medical object to reach a right diagnosis and ignored them as a human being. There was dominant role of doctors and marginal nonverbal communication skills were displayed by the residents in the form of lack of social touch, and appropriate eye contact due to documenting notes. A brief non-medical interaction for rapport building at the beginning of interaction was missing and there was lack of child involvement.

**Conclusion:**

Paediatric postgraduate residents were polite while communicating with parents and child but lacking in good nonverbal communication skills. Communication pattern in our study was mostly one-way showing doctor’s instrumental behaviour and ignoring the child participation.

## INTRODUCTION

A caring relationship is established by professional conversation between patient and treating doctor. In primary care consultation, physician and patient communication is an important component of patient care. Treating physician mostly focus on the technical and biomedical aspect of the case and they generally ignore patients’ own feelings and values. It is noticed that doctors’ clinical practice reflects their moral responsibility to improve patients’ health, while the human and personal dimensions of patients’ suffering are overlooked.^1^ Medical care is based on interpersonal interactions including emotional and related phenomenon like nonverbal behaviour.^2^ The critical factors predicting the quality of care and patient safety are physician empathy and relational skills.^3^ People judge their healthcare standard on its nontechnical aspects like healthcare provider’s communication and soft skills.^4^,^5^ Poor communication is an important cause of adverse events in health care system, resulting in medical errors. Patient safety is at risk when health care provider is not communicating properly due to lack of critical information, misinterpretation of information endangering patient safety.^6^,^7^

During last few decades the importance of good doctor-patient communication has been increasingly emphasized with inclusion of communication skills workshop in postgraduate training program.^8^ In this era of increasing malpractice litigation, doctors need to examine their communication skills. Video recordings provide a rich data source for health care providers’ clinical performance which cannot be analyzed by observation alone. We planned this study for the purpose of analyzing in detail all verbal and nonverbal communication skills of paediatric postgraduate residents in patient-parent-doctor clinical encounter.

## METHODS

The study was conducted at The Children’s Hospital and The Institute of Child Health (ICH) Lahore from August 2016 to December 2016. This was a qualitative exploratory research, conducted through video recording. Approval for the study was taken from the Institutional Review Board of ICH, Lahore. Purposive sampling was done with inclusion of 12 postgraduate residents (PGR) of both sexes working in the ICH who had attended the mandatory communication skills workshop offered by CPSP and are in last year of training. Data (video recording) was collected in the morning class from 8.00 am-9.00 am on every Wednesday (long case discussion day) in the hospital auditorium when the pediatric postgraduate trainees was taking history and doing examination. Data (12 video recording) was collected by trained person in audiovisual department of hospital while the postgraduate trainee was interacting and examining the patient. Postgraduate residents and patients were informed about the purpose of video recording. Analysis was done by researcher and a trained clinical teacher.

Our qualitative analysis was a thematic description which aided in organizing the content and arriving at a narrative description of the postgraduate resident’s behavior during the entire clinical encounter. We identified codes through open coding process i.e. working with categories of interest and then selective coding from interconnection of these categories. Finally, by merging of open codes and axial codes sub-categories were arranged under a theme. Transcription (Notes) of video recording was done by the primary researcher. Paediatric Consultation Assessment Tool (PCAT) and Arizona Clinical Medical Interview Rating Scale (ACIR)were used for priori coding of interest.^9^,^10^ These instruments/tools helped in priori coding. The categories of our interest were made explicit which helped the clinical teacher to use them and obtain substantially the same results. We viewed all the video recording and translated the doctor patient conversation into English language. One videotape was coded by the both researchers independently and discussed to confirm the themes.

## RESULTS

**Fig.1:**
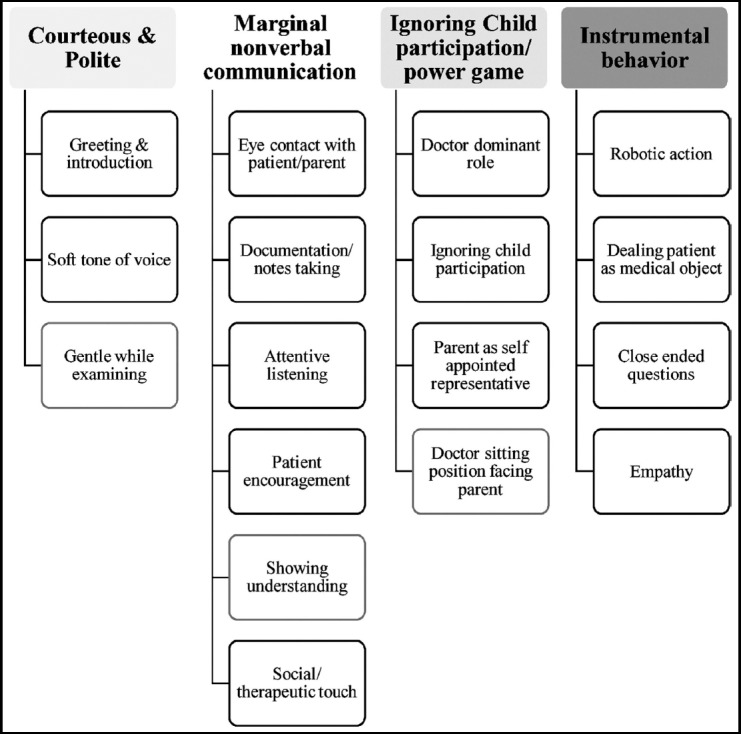
Hierarchy of themes and subthemes.

Initially open coding based on important categories of interest generated a total of 36 codes and then through axial and selective coding these was condensed to 17 subthemes. Finally, by merging of open codes and axial codes sub-categories were arranged under four major themes. These themes seem to be discrete, but there is considerable overlap among them. The hierarchy of themes and subthemes are given in Fig.1.

### Courteous and polite

A doctor actively sets the stage during first minute of interaction for an effective interaction. All clinical encounters began with doctors’ greeting such as “Hello” and doctor introducing himself to the parents of the child. All doctors opened the session in very polite, well-mannered and courteous way in good tone. They greeted the parent and few greeted the child as well and explained the purpose of the interview. PGR: *“Hi, I am Dr. XYZ and want to know about your child’s illness.”* Along with the soft tone in voice the softness in touching the child was quite obvious. PGR touched the face of the child with love softly and asked *“Please take off your shoes and lie down.”*

### Marginal non-verbal communication skills

Health information technologies like paper-based charts or computer interfere in doctor-patient face to face interaction. The trainee used paper-based charts to take notes. Resident used eye contact but mostly they were concerned with the record keeping only. The resident paid less attention to making eye contact with the children in their clinical encounter. PGR looking toward the mother and asked: *“What is your child’s name?”* Mother: also, looked toward the doctor and answered *“Ayesha”* PGR Looked toward the mother for short duration and then while writing the name of the child again asked while gazing toward the paper *“why did you bring your child to the hospital”* Mother: also, gazed towards the paper and answered, *“She had fever”* and then stopped talking as the PGR was not looking in her eyes. One resident who was not taking notes in the clinical encounter and asked all questions with directly gazing to mother’s eyes. She often used affirmative head nodding. She used words “*ok”, “yes” “right”* frequently to show good understanding and concern for the patient and smiled during the interaction. Resident encouraged the parent when she hesitated in answering the question. PGR: “*Please tell in detail about the insulin advised to you without being emotional and be relaxed”* As the mother looked anxious, doctor gave her a reassuring smile and the mother started talking again. Prompting the patient to continue by repeating the patient’s last phrase in a question tone is another method of encouraging the patient. Few residents used this technique. PGR: *“You told that she sometimes had low sugar level, what does she feel during that phase of low sugar and what action you take to improve that low sugar level state?”* PGR: *“Can you please tell in more detail what happened to the child when she became unconscious, you told that she suffered from measles rash, were there any other problems at that time?”* Mother: *“yes she was in the habit of drinking large amount of water and she also passes urine frequently and her mouth becomes dry often”* The resident talked in the same language of that of the patient and showed good understanding of patient’s ideas. While analyzing the nonverbal behavior of the resident it was found that the touch was only therapeutic and there was not a single occasion of social touch. PGR nodded head on and off while listening to the answers. The parent became emotional during interaction and started crying but the resident didn’t offer social touch to calm her down.

### Ignoring child participation and power game

Doctors and patients often experience a power struggle in their communication and social interaction. There was greater number of resident’s conversational acts reflecting clinicians’ dominance. The patient’s role was passive during medical consultation due to low education and lack of health literacy. Beyond the initial segment of exchange of greetings, the relationship shifted as the power difference between doctor and patient where doctor acted as an expert and patient as lay person.

### PGR

“*My name is Dr. XYZ and I want to know about your child’s disease. “I’ll ask few questions regarding your child illness”*. *“What has happened to your child for which you brought him to the hospital?”* All these enquiries are mostly framed as open ended questions and were meant for encouraging patient participation and communication in clinical encounter. After the initial one or two minutes the resident switched towards more close ended questions. All residents interrupted their patients in first few seconds of clinical encounter. The child’s participation was negligible. The time scores were higher for instrumental questioning than for social or emotional issues. PGR: “*How much activity your daughter can perform?”* “*Can she go to toilet herself?”* Father: *“Initially she was normal and was going to school but now bed ridden from last three months”* PGR: *“Does she have only nocturnal bed wetting, or there is continuous dribbling of urine”* There were only few instances of interruptions by the parent at their own. Father: “*Can she start walking again at her own”* PGR: *“Yes she can, but currently she is bed ridden due to her severe illness.”*

Majority of the residents did not involve the child in the discussion except for only once or twice during whole session and mostly in the form of social talk or a brief question. In most of the videotape analysis the interaction was doctor-patient dyads, not triadic with little involvement of children. PGR: “*Aqsa, in which class do you read?* Patient: “*class 5”*PGR: asked the mother about the type of insulin being given, mother stopped for a while and looked towards her daughter in inquisitive way and asked “*R*”. The girl (patient) prompted *“NR”* and mother then repeated her daughter’s words *“NR”* Now the resident directed the turn towards the child and involved her in conversation and asked: “*Whether you yourself do mixing of insulin and inject it yourself*” After that she again directed the turn towards the mother and asked “*How do you store insulin and where?”* PGR addressed mother and asked *“Before this episode of illness and unconsciousness which type of insulin were you giving to Aqsa?”* Mother was unable to answer properly and the girl interfered by telling that “*she was taking 70/30 insulin morning and evening.”* though the turn was directed towards mother. In most of the clinical encounter even if the question was directed towards the child, the adult interrupted the doctor and child speaking resulting in doctor-adult dyad with the child being excluded. Parents in most of the encounter acted as self-appointed representative of the child. PGR directed the turn towards the patient and asked PGR: “*did you suffer from breathlessness while walking?”* Before the child can answer mother said *“yes she sometimes becomes breathlessness while walking”* PGR: turn directed towards the child *“When you suffered from similar illness one and a half year back, were you able to walk or go to the toilet?”* Mother: acted as self a representative translator of her child and said: *“no, she couldn’t walk at that time and we had to carry her to the toilet”* In three of clinical encounter the child was relatively more sick and was lying on the bed and doctor was sitting in a position facing towards the parent so there was very little interaction with the child.

### Instrumental behaviour

All the residents displayed same type of robotic action started with self-introduction followed by organizing their copy pen, and starting questioning about the disease process. Some looked towards patient and some initiated session while directing their gaze to their documenting papers. None of the resident tried to give some time to the child to build rapport or develop a good interpersonal relationship with the child or the parent. Majority treated the patient as a medical object and ignored them as a human being. The main aim of their communication was to reach a right diagnosis. Asking sufficient questions in short period of time and asking right questions was the main aim of residents which resulted in the imbalance between close ended and open ended questions. PGR: *“Can you please tell for what reason you brought your child to the hospital”?* Mother: *“we came for central line placement and fistula formation for hemodialysis”*. PGR: *“What was the age of child when disease diagnosis was made or since when patient is diagnosed as a case of chronic renal failure?”* After that resident started asking close ended questions, PGR: *“Was there history of urinary dribbling?” “Was he ever continent for urine?”*. *“Was there any lump on the child’s back?” “Any history of bed wetting at night?”* These all questions were of the type in which answer would be yes/no. The residents’ behavior was more instrumental versus affective and was more towards the cure as compared to care side of interaction. In all the clinical encounters the patient was taken as a case not as a person. PGR was taking notes and was busy in documenting the history and there was a gap of silence. Mother: took the opportunity of silence and said “*My daughter has history of falling hair.”* PGR: looked blankly toward the mother and didn’t answer or showed any interest in mother’s concern of falling hairs and asked a new question totally irrelevant to the mother’s concern. *“Is there any history of ulcer formation on her feet?”* Almost all of the conversational acts were one way from doctor to parent and revolved around the disease process.

## DISCUSSION

In clinical practice communication is considered as a core skill. The question, “are you a good doctor?” is frequently asked by patients, government and media personnel. To meet the expectations of patients, a good technical skill as well as interpersonal skill is essential along with ethical practice and professionalism.^11^ We found in general that postgraduate residents were very polite to the parents of the child patient. Our results are supported by various studies in which doctors showed very courteous towards their patients.^12^ Communication is not merely verbal in form but is more affected by body language, attitude and tone of voice.^6^ Voice tone has a vital role in communication style and patient’s perception and satisfaction. Only by analyzing tone of voice, any analyzer who is blind towards the doctor can identify those surgeons who had a history of malpractice claims.^13^ A patient’s quote from a qualitative research done in Canada, *“You can hear it in their voice and you can see it in their tone. They actually care how you are feeling” “Their demeanor, their body language, how they speak to you, their tone of voice, the eye contact that they make with you. I think those are primary indictors of compassion”*.^14^

Our study results showed that the doctor’s actions were carried out with pauses in communication and in most of the videotapes it was quite evident that resident was more involved in record keeping than eye contact with the patient or the parent. Our results are consistent with a previous study done by Montague, et al, in which doctors used paper based charts for notes and relationship of mutual gaze and empathy scores were analyzed and it was shown that percentage of eye contact is an important indicator of patient’s perception of empathy.^15^

Understanding the cultural aspects of modesty is very important for doctors when they deal with females of Asian origin.^16^ Our research finding showed lack of social touch in case of emotional situations as doctor ignored the importance of social touch when a mother started crying on her child’s disease. A study published in British Journal of General practice described that most patients believed that expressive touch was acceptable especially in situations of distress.^17^ A result of a study done by Fahad in Pakistan showed that nonverbal behaviour helps in strengthening doctor-patient relationship as patients stated that they appreciate positive touch and eye contact from their physician. This study regarding the use of comforting touch to patients by physicians ensures this as 82.5% of respondents said that they wanted sympathetic touch especially in distressing situations.^18^

Our most of the parents accompanying the child were mothers and mostly from low social class on social history gathering. This resulted in lower patient control over communication and change in the dynamic of consultation to more of a power game by doctor in dominant position. Similarly in a study by Evelyn it is shown that both the patient’s social class and his/her communication style influences doctor-patient communication.^19^

Extensive literature search on communication in paediatric consultation and this issue of ignoring child’s participation in clinical encounter show results in which child participation was very much lacking.^20^,^21^ The results of our study showed similar findings that children were not treated as active participants in their medical encounters and if they are involved, parent took them as self-appointed representative of their child and often stop the child to participate resulting in shift of the participant role in medical consultation.

In our study the communication style was mostly one way in which doctor was in leading position. Same type of results was documented by Claramita et al. which proved that in South East Asia a more one-way style or paternalistic with little input from patient is seen.^16^ Our results illustrate that residents spent most of their time in medically related talk and spent almost negligible time in non-medical talk of rapport building. Our results were supported by a study by Kain et al. in which health care provider has spent largest proportion of their time in medical related talk and relatively small percentage of time was spent in non-medical talk or talking to children.^22^

While communicating with the parents the residents sometimes ignore the parental concern as they consider them irrelevant to the main problems thus undertaking existential filtering. Several qualitative studies and reviews from different corners of the world have reported similar concerns of ignoring existential concerns by various researchers.^1^ Existential filters often take the focus away from the patient’s concerns and focus only the practical issues.

### Limitations

Our study was only observational and use of video cameras may have influenced behaviour of both patients and doctors. These results cannot be generalized to all postgraduate residents or doctors, as communicating with patient in a class room setting may be different from their routine communication in their daily practice.

## CONCLUSION

The doctor-patient interaction during clinical encounter in pediatric setup is a complex relationship involving the doctor, child patient and parent which is being restricted to the dyadic interaction between doctor and parent by their non-supporting role. Central values underpinning patient-centered care, i.e., equal sharing of power when interacting with the patients in the clinical encounter is lacking. Mechanical manner of handling the patients and shift from talking respectfully to reducing them to medical object by letting the dialogue drift into documentation or physical examination is a common practice. Video based research is a promising method in primary care clinical encounters. It is helpful in detailed analysis of both verbal and nonverbal communication of doctors’ in clinical setting and can yield significant benefits by understanding the practice of residents and generating intervention for improvement in communication.

### Authors’ Contribution

**AB:** Main author.

**RAK:** Critical review, editing and suggestions.

**UJ:** Co-researcher and Clinical teacher.

**AWR:** Review & Final approval of the version to be published.
